# Reintegrating the Human in Health: A Triadic Blueprint for Whole-Person Care in the Age of AI

**DOI:** 10.3390/ijerph23040426

**Published:** 2026-03-29

**Authors:** Azizi A. Seixas, Debbie P. Chung

**Affiliations:** 1 Department of Informatics and Health Data Science, University of Miami Miller School of Medicine, 1120 NW 14th Street, Miami, FL 33136, USA; 2 Department of Psychiatry and Behavioral Sciences, University of Miami Miller School of Medicine, Miami, FL 33136, USA

**Keywords:** integrated care, digital health, artificial intelligence, health systems strengthening, health equity

## Abstract

Modern healthcare remains structurally and conceptually fragmented, with profound clinical and policy implications. At its root lies an ontological fracture: the prevailing biomedical model reduces patients to discrete biological systems (organs, biomarkers, and symptoms) detached from the psychological, social, and ecological contexts in which health and illness are experienced. This is compounded by epistemological fragmentation, where medical knowledge is compartmentalized into increasingly narrow specialties, limiting holistic understanding. These philosophical divisions manifest in downstream operational, informational, financial, and policy dysfunctions duplicative testing, misaligned incentives, disconnected care pathways, and population health failures. To address these multilevel fractures, we propose a unified architecture grounded in three interlocking components. First, the Precision and Personalized Population Health (P3H) framework offers a principle-based realignment toward care that is integrated, personalized, proactive, and population wide. P3H addresses the conceptual shortcomings of fragmented care by focusing on the full human trajectory across time, systems, and determinants. Second, General Purpose Technologies including artificial intelligence, biosensors, mobile diagnostics, and multimodal data systems enable the operationalization of whole-person care at scale, especially in low-resource settings. Third, the AI-WHOLE policy framework (Alignment, Integration, Workflow, Holism, Outcomes, Learning, and Equity) provides governance principles to guide ethical, equitable, and context-specific implementation. We argue that this triadic blueprint is particularly critical for Global South nations, where the lack of legacy infrastructure offers an opportunity for leapfrogging toward integrated, intelligent systems of care. Early models illustrate how policy-aligned, technology-enabled care rooted in whole-person principles can yield improvements in continuity, cost-efficiency, and chronic disease outcomes. This manuscript offers a systems-level strategy to overcome fragmentation and reimagine healthcare delivery, not only by refining clinical tools, but by redefining what it means to care for the human being in full.

## 1. Introduction: The Problem of Fragmentation in Modern Medicine

Modern healthcare is paradoxically advanced and sophisticated, yet fractured and fragmented. Despite extraordinary advances in biomedical science, patients are still routinely interpreted and treated as collections of discrete biological systems, symptoms, and biomarkers rather than as integrated human beings. This is not merely a coordination failure; it reflects deeper philosophical fragmentations that shape what clinicians notice, measure, and act upon. Ontological fragmentation reduces the person to organ systems and measurable proxies (e.g., HbA1c, ejection fraction, and PHQ-9), while epistemological fragmentation separates legitimate medical knowledge from psychological suffering, lived experience, culture, and the social and ecological conditions that structure risk and recovery. These fragmentations are reinforced by clinical training, specialty-based credentialing, and research pipelines that privilege narrowly bound outcomes [1]. The result is not simply specialization, it is a patterned clinical gaze that struggles to perceive the patient’s gestalt: the interacting biological, psychological, and social realities that co-produce illness trajectories.

Once entrenched, these philosophical fractures predictably cascade into the operational architecture of health systems. They surface as operational fragmentation (disjointed workflows, inconsistent follow-up, and weak coordination), informational fragmentation (scattered records, incompatible systems, and uneven governance), financial fragmentation (misaligned incentives that reward volume over synthesis and prevention), and policy fragmentation (separate mandates and funding streams for physical health, mental health, public health, and social services). The cumulative effect is a delivery environment in which no actor is structurally positioned or reimbursed to assemble the whole-person narrative over time. Empirically, contemporary care patterns reflect this diffusion of responsibility where Medicare beneficiaries distribute care across multiple clinicians and practices, limiting any single physician’s ability to own the whole story and constraining reforms that assume stable attribution and continuity [2]. Over time, outpatient care has shifted toward more specialist encounters with more unique physicians, expanding the coordination burden placed on primary care and increasing the likelihood that no clinician synthesizes the full clinical and contextual picture. Meanwhile, informational fragmentation extends beyond interoperability failures: at the population level, patients often lack access to reliable, personalized health information while misinformation spreads at scale, weakening prevention, adherence, and trust. High care fragmentation is associated with increased mortality, missed diagnoses, avoidable hospitalizations, polypharmacy, and substantial waste in healthcare spending [3].

These divisions are clinically consequential because they produce a predictable workflow of decomposition, where “what is wrong” is broken into parts and treated in parallel rather than synthesized. Consider a patient presenting with fatigue, depressive symptoms, and poorly controlled diabetes. Ontological fragmentation converts this lived experience into discrete clinical objects; epistemological fragmentation routes the patient into separate domains of expertise; operational fragmentation yields unconnected encounters and care plans; informational fragmentation blocks context and longitudinal meaning; and financial and policy fragmentation discourages the integrative work required to address syndemic interactions and upstream determinants. The consequences are not only experiential but measurable: fragmented ambulatory care has been associated with greater acute care utilization and costs, including unnecessary procedures and testing, more emergency department use, and more hospitalizations. At the system level, variation in how tightly clinicians “work together” (i.e., networked teamwork) is associated with downstream outcomes after major procedures, including readmission and mortality, evidence that fragmentation is not merely conceptual but operationally expressed in avoidable harms [1].

The literature offers important partial solutions, yet they are often layer-bound. Integrated care and delivery reforms tend to focus on operational coordination without directly correcting the ontological and epistemological drivers that continuously recreate siloed reasoning. Precision medicine and “big data” initiatives, conversely, can intensify measurement of biomedical signals without ensuring whole-person synthesis across behavioral, social, and environmental context [4]. Digital health strategies frequently add tools without re-aligning incentives and governance. Against this backdrop, this manuscript advances a distinct improvement strategy, a triadic architecture that explicitly maps interventions to the fragmentations they are designed to repair. Specifically, (1) Principles (Precision and Personalized Population Health [P3H]) address philosophical fragmentation by re-grounding care in whole-person synthesis focusing on personalized, proactive, population-based, and human-centered health care; (2) Products (General Purpose Technologies) address operational, informational, and financial fragmentation through scalable data integration, decision support, sensing, and workflow redesign; and (3) Policies (AI-WHOLE) address policy fragmentation by aligning governance, accountability, and equity with person-centered AI-enabled care. In doing so, we move beyond aspirational calls for integration by specifying what must change, where, and how, including the implementation implications for multimodal data integration, synthesis-oriented workflows, and evaluation metrics that capture continuity, outcomes, equity, and whole-person experience.

The need to create urgent solutions for fragmented healthcare is urgent as health systems face growing pressure to deliver better outcomes without proportional increases in spending. The Organization for Economic Co-operation and Development (OECD) projections suggest that public health spending will continue to rise as a share of GDP, yet budgets are increasingly constrained by other national priorities, making value and efficiency unavoidable policy goals [5]. In the United States, high health expenditures and persistent affordability problems make strategies that rely on additional spending increasingly unrealistic. At the same time, the burden of chronic, complex diseases is rising and increasingly affecting people earlier in life, expanding the number of years patients spend navigating multiple conditions, clinicians, and care settings [6,7,8]. This combination of fiscal constraint and growing clinical complexity makes fragmentation more consequential and more expensive, because preventable utilization, duplicated testing, missed risk signals, and poorly coordinated long-term management accumulate over decades rather than episodes [9]. Artificial intelligence and other general-purpose technologies (GPT) are therefore needed now as enablers of whole-person care because they can integrate multimodal clinical, behavioral, and contextual data into actionable care pathways, support continuous management beyond visits, and reduce avoidable utilization when implemented with accountable governance. The central question addressed in this manuscript is whether these technologies can be governed as a whole-system operating model that reduces fragmentation and improves function, equity, and long-term outcomes, rather than delivering isolated efficiency gains within an already fragmented system.

Several established frameworks motivate this manuscript but leave a persistent implementation gap. The World Health Organization Integrated People-centered Health Services framework calls for redesigning health systems around people rather than diseases and explicitly identifies fragmentation as a threat to sustainability, yet it does not specify how AI and general-purpose technologies should be governed as enforceable policy for whole-person representation and pathway accountability. Learning Health System approaches align science, informatics, incentives, and culture for continuous improvement, but typically remain meta-level and do not operationalize minimum biopsychosocial constructs, procurement standards, or pathway certification that would prevent point solutions from reproducing fragmentation [10,11]. AI governance and assurance efforts strengthen evaluation and reporting, yet they often focus on properties of AI systems rather than the longitudinal care pathway as the unit of accountability. AI-WHOLE is positioned to bridge these traditions by binding precision and personalized population health (P3H) clinical logic to general-purpose technologies through policy levers that make whole-person care implementable, measurable, and scalable.

## 2. Principles, Products and Policies to Address Fragmentation

Addressing fragmentation in healthcare requires more than incremental coordination fixes; it demands a paradigmatic redesign that operates across three interdependent layers, principles, products, and policies because the most visible failures of modern care (disjointed workflows, siloed data, misaligned incentives) ultimately arise from deeper philosophical fractures in how the patient is understood and how clinical knowledge is organized. When ontological fragmentation reduces patients to organ systems, symptom clusters, and biomarkers, and epistemological fragmentation splits legitimate medical reasoning from psychology, social context, and lived experience, the downstream system is almost destined to reproduce operational, informational, financial, and policy fragmentation. The response must therefore be architectural describing how: (1) the P3H framework can repair the philosophical fragmentation in medicine; (2) general-purpose technologies (GPTs) supply the enabling capabilities that make those principles executable in day-to-day operations; and (3) AI-WHOLE supplies the policy and governance scaffolding that prevents the system from reverting to siloed practice and ensures that whole-person intent becomes durable, measurable practice.

At the principles layer, the precision and personalized population health (P3H) framework functions as a corrective to ontological and epistemological fragmentation by requiring that care be personalized, proactive, population-based, and human-centered [12]. P3H operationalizes integration not as a vague aspiration, but as a clinical and system requirement where care plans must synthesize biological, behavioral, social, and environmental drivers of risk and recovery, rather than treating them as externalities. This directly counters reductionist clinical reasoning by making context part of the clinical object, so upstream determinants (e.g., housing instability, food insecurity, and trauma exposure) are treated as causal inputs to diagnosis, prognosis, and intervention rather than as background. Systems that fail to integrate these domains predictably experience more medication errors, diagnostic delays, and preventable hospitalizations [13]. P3H also reframes personalization as more than genomics or pharmacology; it centers patient values, preferences, cultural context, and lived constraints as determinants of what the right treatment means and whether it can be sustained [14,15]. Further, by insisting on proactivity, P3H shifts the system from late-stage disease management toward risk anticipation, prevention, and early intervention, particularly for chronic conditions whose trajectories are strongly shaped by behavior and environment [16]. Finally, P3H’s human-centered emphasis forces coordination to be designed rather than assumed, including key components such as longitudinal accountability, shared decision-making, and cross-sector teamwork as required features of care delivery. Without interoperability, shared accountability structures, and aligned incentives, even well-designed clinical innovations degrade into siloed implementation [17]. In this way, P3H is not simply another care model; it is a philosophical and operational reset that makes whole-person synthesis the standard for clinical reasoning and health system design.

Principles alone, however, cannot reorganize practice at scale. The product/technology layer specifies how P3H becomes executable through general-purpose technologies (GPTs) including artificial intelligence, multimodal sensing (biosensors and wearables), mobile platforms, computing, and advanced analytics that can represent the whole person longitudinally and translate whole-person representation into clinical and public-health action [18]. This requires a modern data stack that links person-level information across settings and time, including structured clinical data from the electronic health record (HER) and claims, unstructured clinical narratives, patient reported outcomes, and contextual signals such as social, environmental, and behavioral factors, and when feasible, omics and imaging. AI then functions primarily as a synthesis infrastructure that converts notes into usable signals, integrates multimodal data, and supports proactive outreach, prevention, and escalation. Importantly, this layer can directly reduce operational and financial fragmentation by standardizing and continuously updating risk stratification and care pathways, which reduces duplicative testing, prevents conflicting care plans, supports team-based decision making, and shifts resources toward prevention and longitudinal management. For example, predictive models can identify patients at high risk for hospitalization by combining clinical history with contextual vulnerabilities such as social isolation or housing instability, enabling earlier interventions that are both clinically meaningful and cost-saving [18]. In short, GPTs are the “implementation engines” that convert whole-person intent into scalable workflows provided they are embedded into clinical operations, not deployed as parallel tools.

Finally, whole-person care cannot become the norm without a policy layer that aligns incentives, governance, and accountability. The AI-WHOLE (Alignment, Integration, Workflow, Holism, Outcomes, Learning, and Equity) framework provides an actionable policy architecture for operationalizing person-centered and AI-enabled care at scale. It translates ethics and equity from aspiration into governance requirements that (1) align technology with care goals and public values, (2) set standards for data exchange and stewardship, (3) embed tools into workflows that promote synthesis rather than separation, (4) define expectations for holism including psychosocial and contextual inputs, (5) require outcome measurement with equity stratification, (6) operationalize learning through continuous monitoring and improvement, and (7) establish safeguards that prevent exclusion. Critically, AI-WHOLE targets policy fragmentation that has long impeded integrated care by requiring cross-sector coordination in reimbursement, regulation, and data governance. Payment systems must shift from volume-based incentives toward value-based structures that reward continuity, prevention, and coordination; regulations must support secure data sharing, transparency, and community participation, and system-level vehicles such as Accountable Care Organizations (ACOs), social health insurance reforms, and national digital health strategies must be explicitly designed to institutionalize whole-person synthesis rather than episodic disease management [19]. When deployed together, these three layers, P3H principles, GPT-enabled products, and AI-WHOLE governance, form a coherent mechanism for replacing fragmentation with whole-person medicine that is clinically actionable, operationally scalable, and politically sustainable.

## 3. The P3H Framework

The Precision and Personalized Population Health (P3H) framework offers a comprehensive blueprint for reengineering fragmented healthcare systems into cohesive, person-centered ecosystems. Grounded in the principles of whole-person care which integrates biological, psychological, social, and environmental determinants, the P3H model structures care delivery across seven interdependent phases: Discovery, Awareness, Avoidance, Access, Assessment, Acceptance, and Adherence (See Table 1). These stages function not as isolated interventions but as a continuous, adaptive system that aligns personalized medicine with population health goals. The deployment of general-purpose technologies (GPTs) including artificial intelligence (AI), advanced biosensors, and molecular diagnostics operationalizes each phase, transforming healthcare from episodic symptom management into a learning system that evolves with the individual and community. By unifying clinical data, behavioral context, and population-level trends, P3H not only improves health outcomes but redefines healthcare as a relational, anticipatory, and dynamically responsive enterprise.

In Discovery, machine learning algorithms integrate genomic, phenotypic, and environmental data to uncover novel biomarkers and causes of disease and treatment pathways. Awareness is enhanced through personalized risk profiles generated from EHRs, wearables, and behavioral data, improving patient engagement and early detection. Predictive analytics in Avoidance (prevention medicine) guide targeted prevention strategies for chronic disease, while AI-supported telehealth and mobile diagnostics in Access improve equity by expanding care to underserved populations particularly in low-resource settings. In Assessment, AI tools such as computer vision and natural language processing (NLP) increase diagnostic accuracy and reveal comorbidities otherwise missed in siloed data. Acceptance benefits from AI’s ability to anticipate treatment hesitancy, supporting shared decision-making, while Adherence is reinforced by real-time monitoring tools and feedback loops that personalize ongoing care. Together, these capabilities allow P3H to unify individualized precision care with population-level strategies, creating a scalable model that treats not just disease, but the whole person biologically, psychologically, socially, and environmentally. By integrating AI across all seven phases, P3H becomes an intelligent, learning healthcare system that evolves with the patient. This approach enhances clinical effectiveness and promotes relational, personalized, and equitable care fusing cutting-edge technology with the timeless goals of human-centered medicine.

The Precision and Personalized Population Health (P3H) framework is an end-to-end antidote to the current fragmented continuous health pathway that intentionally links discovery, risk awareness, prevention, access, diagnosis, early treatment engagement, and long-term adherence. In doing so, P3H directly counteracts the philosophical fragmentations described earlier by rejecting an ontologically part-based clinical focus by requiring integration of biological, behavioral, social, and environmental drivers of health, and countering epistemological silos by forcing clinical knowledge to be synthesized across domains rather than delegated to disconnected specialty logics. Practically, P3H operationalizes whole-person care by converting context into care actions: risk identification is paired with prevention supports, patient goals and preferences shape treatment selection, and longitudinal monitoring is treated as a core clinical function rather than an afterthought. This approach is consistent with precision-population health applications that deliberately combine community context, individualized risk stratification, and tailored interventions to address chronic disease drivers upstream (e.g., sleep–cardiometabolic risk) rather than only managing downstream complication [15].

In practice, P3H is implemented when a health system (or a tightly integrated network) builds infrastructure that can (1) generate or absorb discovery signals (e.g., omics, imaging, and EHR phenotypes), (2) translate those signals into patient-level and population-level risk awareness, (3) deliver targeted prevention, (4) remove access friction, (5) strengthen diagnostic synthesis, and (6) support early treatment engagement and long-term adherence through monitoring and feedback. Turkey provides a strong population-scale example of P3H-enabling infrastructure via the national personal health record e-Nabız has been used at very high national penetration (reported to be approximately 82% of the population), and it explicitly supports prevention, self-management, and reduced duplication through national sharing of laboratory/radiology data [20]. More directly aligned with the Awareness, Avoidance, Assessment, Adherence arc, Turkey’s national Disease Management Platform (DMP) is a family physician–centered, nationally deployed system that integrates risk assessment, decision support, patient self-management plans shared into e-Nabız, and population tracking tools.

Importantly, although P3H is rarely implemented as a labeled national program, its elements have repeatedly produced measurable gains when health systems build discovery-to-adherence pathways rather than isolated episodes of care. A widely cited example is Kaiser Permanente Northern California’s large-scale hypertension program, which used population registries, standardized treatment pathways, team-based workflows, and proactive outreach, core P3H mechanics, to raise hypertension control from 43.6% to 80.4% (2001–2009) and was associated with substantial improvements in cardiovascular outcomes in the covered population [21]. Similar system designs population risk identification, protocolized escalation, and ongoing monitoring have been highlighted as scalable population-health management strategies precisely because they turn fragmented encounters into a coherent longitudinal pathway. These kinds of results strengthen the manuscript’s core claim: whole-person medicine becomes feasible when health systems are engineered as learning pathways, not as disconnected specialty transactions.

Another example and more compelling use case is seen in Turkey’s national Disease Management Platform (DMP) system, which provides primary care embedded chronic disease screening and management to standardize risk assessment, early detection, follow-up, and patient self-management. DMP is built around four operational capabilities: (1) screening and risk assessment (e.g., periodic and precise population health diabetes and hypertension screening by age/risk criteria), (2) disease progress monitoring for diagnosed patients with guideline-aligned care plans (labs, risk recalculation, individualized goals, medication suggestions, referral prompts), (3) patient self-management support by pushing care plans, education, and lifestyle recommendations into Turkey’s national personal health record (e-Nabız), and (4) panel-based population tracking (family medicine physicians typically manage approximately 2000–4000 patients) that supports overdue lists, performance dashboards, and SMS outreach [22] As of September 2023, the platform had been used by >25,000 clinicians, delivering >73 million screening/monitoring encounters for >16 million individuals, with screening contributing to large numbers of newly identified cases (e.g., approximately 145k hypertension, 490k diabetes, >500k high cardiovascular risk, and >3.5 million obesity), and enabling large-scale assessment of treatment goals across millions of follow-ups [22]. This is a concrete demonstration of how P3H can be executed as a national longitudinal workflow.

## 4. General Purpose Products and Technologies as a Solution for Fragmentation

General-purpose technologies (GPTs), particularly AI, connected sensor ecosystems, and cloud-based data infrastructure, function as the products layer that makes whole-person care operational rather than aspirational. Where fragmentation persists because clinical reality is divided across encounters, sites, and domains, GPTs can reconstruct the patient as an integrated, longitudinal signal by capturing multimodal data (physiological, behavioral, contextual), integrating it across settings, and converting it into actionable insights for patients, clinicians, and health systems. In practice, this means moving beyond episodic, facility-bound snapshots toward continuous, person-in-context measurement: wearable and ambient sensors generate patient-generated health data (PGHD) that can surface symptom burden, functional status, sleep-wake cycle, mobility, stress physiology, and treatment side effects, components often invisible to conventional workflows yet central to whole-person care.

The strongest evidence that GPTs can reduce downstream harms of fragmentation comes from deployments that close the loop between sensing, analytics, and response. Digital sensor-driven alerting and monitoring systems (a blend of sensors, connectivity, and analytics) have been associated with improved clinical outcomes in aggregated evidence. For example, a systematic review and meta-analysis of digital sensor alerting systems reported associations with reductions in mortality and hospitalizations, suggesting that continuous signals can enable earlier detection and intervention than episodic visits alone [23]. In heart failure, multi-sensor wearable monitoring paired with analytics has been studied as a mechanism to detect impending decompensation and support earlier outpatient action precisely the kind of workflow that converts disconnected episodes into a coordinated trajectory [24].

Complementing sensor-based GPT, AI methods (e.g., machine learning risk prediction, NLP synthesis of notes, and multimodal fusion of PGHD with EHR and claims) serve as the integrative cognition layer that can reconcile cross-domain signals medical, behavioral, and social into coherent risk profiles and tailored care actions, including safety surveillance and error prevention across complex care pathways [25,26]. Cloud computing infrastructure can determine whether whole-person data becomes shared clinical reality or remains siloed artifacts. Health information exchange (HIE) and cloud-enabled medical records access directly address informational fragmentation and have repeatedly been associated with reductions in duplication and inefficiency. For example, observational work has found that faster HIE access in emergency care is associated with shorter length of stay and lower charges, consistent with fewer redundant evaluations [27]. Finally, because whole-person data are inherently broader (and therefore riskier), privacy-preserving computation becomes part of the product layer where federated infrastructure and learning models enable multi-site model development without centralizing sensitive data, while blockchain-based architectures can strengthen auditability, access control, and integrity across distributed records capabilities that matter when whole-person care requires cross-institutional coordination [28,29].

In short, GPTs can overcome fragmentation when they are deployed as an end-to-end capability capturing whole-person signals, integrating them across settings via interoperable/cloud infrastructure, securing them with privacy-preserving architectures, and using AI to translate complexity into coordinated, timely, person-centered action. The integration of General Purpose Technologies (GPTs) within the Precision and Personalized Population Health (P3H) framework promises to transform not only clinical workflows but the entire healthcare ecosystem. In education, medical curricula must evolve to equip future clinicians with the capacity for systems thinking, AI fluency, and human-centered design. Foundational topics such as data science, genomics, behavioral science, and digital ethics should be embedded into both undergraduate and postgraduate medical training to prepare clinicians for hybrid human-machine teams [30]. This also includes teaching students to critically evaluate algorithmic decisions, integrate multimodal data into diagnosis and treatment planning, and lead ethically sound implementation efforts. Integrated training that breaks down silos between clinical, basic, and social sciences is essential to cultivating physicians capable of delivering whole-person care [31].

## 5. The AI-WHOLE Policy Framework

Health care is entering an AI-enabled era while remaining structurally fragmented where data, accountability, and incentives are distributed across clinicians, payers, sites of care, and vendors, such that tools that perform well locally often fail to improve outcomes longitudinally for the same person. Current AI policies address important components, including data exchange, coverage integrity, evaluation discipline, and payment alignment, but they do not provide a unifying operating policy that makes whole-person care the unit of implementation and accountability. Interoperability and information-blocking policy (e.g., the 21st Century Cures Act final rule) improves the feasibility of accessing and exchanging electronic health information yet does not specify what constitutes a minimally sufficient representation of the person (biology, behavior, context, goals, and function) for AI-enabled care [32,33]. Center for Medicare and Medicaid Services (CMS) guidance appropriately constrains the use of algorithms in coverage determinations but is largely prohibitive rather than prescriptive for designing whole-person, end-to-end care pathways. Deployment governance guidance such as the NHS’s real-world AI evaluation lessons strengthens implementation and learning under routine conditions but does not require that AI be organized around an explicit whole-person clinical logic with measurable longitudinal outcomes. These gaps create a predictable failure mode where AI optimizes tasks while perpetuating fragmentation. A new policy framework is therefore needed that integrates P3H and general-purpose technologies into a coherent, enforceable system of infrastructure, practice, and assessment capable of treating the patient in totality, authentically, pragmatically, and cost-effectively.

Existing AI policies are necessary but incomplete because they govern prerequisites and constraints rather than the clinical-structural requirements of whole-person care. Interoperability policy enables exchange but does not establish a whole-person minimum dataset or mandate structured capture and portability of goals, function, behavioral factors, and environmental context as first-class elements of care. CMS guidance clarifies that algorithmic tools may assist Medicare Advantage coverage determinations only within applicable rules and medical-necessity standards, but it does not specify how to build affirmative pathways in which multimodal determinants are integrated into discovery, prevention, treatment, and adherence decisions [34]. The CMS guidance emphasizes the practical design of real-world evaluations of AI and is explicitly oriented toward implementation, measurement, and learning, yet it remains agnostic to whether wholeness is represented and acted on as a system obligation rather than an aspirational outcome. Reimbursement-oriented policy similarly recognizes that sustainable adoption requires payment models that reward patient and population benefit, but these approaches often price discrete AI services rather than underwriting continuity as an operating model across the patient journey. The consequence is that health systems can meet interoperability, coverage, and evaluation expectations while still deploying AI as point solutions that deepen silos.

The AI-WHOLE Policy Framework is proposed as the missing whole-person operating policy that binds Precision and Personalized Population Health (P3H) to the enabling capacity of general-purpose technologies (GPTs) (See Table 2). AI-WHOLE is positioned not as an ethics checklist and not as a technology taxonomy, but as policy infrastructure designed to prevent new tools from reproducing old fragmentation by hardwiring whole-person requirements into procurement, interoperability, workflow certification, reimbursement, and lifecycle assurance. This distinction is increasingly salient as generative AI tools diffuse rapidly. For example, in a multicenter quality-improvement study across 6 health systems, ambient AI documentation demonstrated measurable benefits in clinician experience where the proportion of clinicians reporting burnout decreased from 51.9% to 38.8% after 30 days of use [35]. In a separate quality-improvement study, informed-consent processes for ambient documentation varied substantially, and patient and clinician perspectives highlighted contextual needs for consent discussions and perceived responsibility for the technology’s use [36]. This juxtaposition illustrates the policy problem AI-WHOLE is designed to solve efficacy in a narrow domain does not ensure whole-person legitimacy, consistent implementation, or accountable longitudinal care.

AI-WHOLE constitutes a policy stack across seven domains (Alignment, Integration, Workflow, Holism, Outcomes, Learning, and Equity) each instantiated through enforceable levers, routine operational practice, and measurable indicators. Alignment is implemented through procurement and governance requirements that mandate a declared clinical purpose mapped to the P3H pathway, named accountable clinical and operational owners, and documented participatory design. Alignment involves participatory co-design with clinicians and communities to ensure tools reflect contextual realities, such as using chatbots in community health clinics to improve medication adherence among low-literacy populations. Integration extends interoperability policy from data can move to the right person-level constructs are represented and computable, requiring structured capture and exchange of patient goals, function, behavioral drivers, and contextual/environmental determinants alongside biomedical data, using privacy-preserving architectures when appropriate; this complements the Cures Act’s exchange intent while adding whole-person content requirements that exchange policy does not specify. Integration enables the unification of multimodal data combining structured EHRs, unstructured clinician notes, and social determinants to build 360° risk profiles, as seen in platforms like Cityblock Health and Unite Us. Workflow requires seamless embedding of AI tools within existing clinical processes, such as natural language processing (NLP) scribes that reduce documentation time [37]. Workflow is operationalized through pathway-level implementation standards, including defined handoffs, escalation processes, human override, audit trails, and safety monitoring; this aligns with the NHS emphasis on real-world evaluation and learning while shifting the unit of implementation from the tool to the audited care pathway. Holism sets minimum biopsychosocial representation standards for decision support so that whole person is present in the logic of care rather than reconstructed after deployment. Holism guides the deployment of AI-enabled health screening via smartphones or wearable detection of social isolation and stress, enabling early behavioral intervention in primary care. Outcomes extend beyond traditional biomedical metrics to include patient-reported outcomes (PROs), goal attainment scaling, and functional status indicators captured via mobile apps and integrated into EHR dashboards. Outcomes ties reporting and payment to goal-concordant and function-relevant measures (patient-reported outcomes and experience, functional status, preventable utilization, total cost), ensuring that whole-person capture is accountable to value and sustainability rather than model discrimination alone [38]. Learning is bi-directional, meaning AI systems continuously retrain local data to improve contextual relevance, while providers are trained in AI literacy to effectively interpret and act on AI-generated insights. Learning requires lifecycle operations, including post-deployment monitoring, drift detection, transparent performance reporting, and predetermined update pathways so tools remain valid as populations and care processes change. Equity is treated as a design imperative, enforced via algorithmic bias audits, inclusive dataset curation, and language localization. Equity mandates stratified evaluation and mitigation plans with time-bound improvement targets to ensure that gains in efficiency or average performance do not widen disparities. For example, AI models developed in partnership with traditionally underrepresented groups can ensure cultural congruence in decision support.

AI-WHOLE policy framework formalizes assessment and economic pragmatism as policy requirements. In aggregate, AI-WHOLE is intended to translate interoperability, coverage integrity, evaluation rigor, and reimbursement alignment into a coherent, enforceable infrastructure for authentic whole-person care, making P3H executable at scale with GPT-enabled systems rather than episodic and fragmented by default. Success is measured using key performance indicators such as reductions in avoidable hospitalizations, improvements in care continuity, and disparity reduction metrics stratified by race, geography, and socioeconomic status. By grounding high-tech innovation in human-centered design and equity, AI-WHOLE provides a practical and ethical framework for transforming fragmented healthcare into an intelligent, inclusive system of whole-person care.

## 6. A Triadic Blueprint for Advancing Whole-Person Care in the Global South

Whole-person care requires an operating model in which clinical intent, scalable tools, and governance are aligned so that the determinants of health such as biology, behavior, and environment are acted on together over time. P3H provides that clinical intent by specifying longitudinal, stage-based care from discovery and risk identification through treatment, adherence, and sustained outcomes. General-purpose technologies (AI, mobile platforms, cloud computing, sensors) provide the execution layer that makes continuous, low-marginal-cost care feasible beyond facility walls. AI-WHOLE policy supplies the missing governance layer that prevents these tools from becoming isolated point solutions by requiring whole-person data representation, pathway-level implementation, and accountable measurement. This three-layer stack is already visible in systems that come closest to integrated, people-centered care, where care redesign is paired with digital infrastructure and rule sets that make coordination routine rather than aspirational.

Brazil’s Family Health Strategy illustrates how the stack becomes real-world practice. The program operationalizes P3H-like logic through multidisciplinary community teams and continuity-oriented primary care, with a large evidence base linking expansion to improved outcomes, including reduced mortality and avoidable hospitalizations [39]. Brazil increasingly reinforced this model with a national primary-care digital backbone through the e-SUS Primary Care strategy, designed to modernize primary care information systems, improve the quality/detail of health information, and support interoperability across municipalities enabling longitudinal tracking, coordination, and continuity at scale [40] The policy implication is the core AI-WHOLE point where whole-person gains do not arise from a single tool but emerge from a care model (P3H logic), enabled by scalable infrastructure (GPT-like capabilities), and stabilized by system-level operating rules.

Rwanda provides a complementary example where whole-person healthcare pathways are strengthened by scalable digital infrastructure and explicit national governance under resource constraints. RapidSMS has been deployed at national scale, with community health workers sending millions of messages and evidence suggesting the platform’s effectiveness depends on being coupled with training, supervision, and operational support, an implementation lesson aligned with AI-WHOLE’s workflow and accountability emphasis. Rwanda also demonstrates how general-purpose logistics can improve end-to-end reliability such as evaluations of drone-enabled blood product delivery which have improved delivery performance and reduced waste relative to ground transport, thus removing a bottleneck that breaks continuity of care even when clinical intent is clear. AI-WHOLE’s added value is to formalize what these exemplars imply which is to make P3H executable with GPTs at scale, where policy must require explicit whole-person constructs (goals, function, behavioral and contextual determinants), pathway certification (handoffs, escalation, auditability), and accountable outcomes (function, preventable utilization, equity).

However, the value and success of the whole-person model is best realized in multi-sector initiatives that are not solely dependent on healthcare to provide solutions. Whole-person health solutions should include healthcare and other adjacent sectors like environmental, education, municipality (e.g., local government), and financial agencies. Two multistakeholder use cases make the whole-person point explicit showing why the operating model cannot rest on clinicians and public health alone. Ahmedabad’s Heat Action Plan coordinated meteorology, municipal services, emergency response, clinicians, and community organizations to shift from episodic rescue to longitudinal risk management (early warning, preparedness, targeted protections), and a peer-reviewed evaluation reported reductions in heat-associated all-cause mortality in implementation years compared with a pre-plan baseline [41,42]. A second use case is London’s Ultra Low Emission Zone (ULEZ) which included transport policy, enforcement, monitoring, and public health goals to reduce exposures to noxious emissions and environmental toxins to improve respiratory and cardiometabolic health [43]. In both cases, the P3H logic (risk identification and prevention across time), GPT-like execution (monitoring, communications, enforcement/logistics platforms), and AI-WHOLE-like governance (alignment across owners, integrated data-to-workflow pathways, measurable outcomes) function together to deliver whole-person care that neither the clinic nor any single sector could achieve independently.

## 7. Implementation Considerations

Institutions will resist AI-WHOLE because it creates economic, social, ethical, legal, and operational friction, and each category has real stakes. Economically, it can disrupt revenue and incentive arrangements by shifting attention from departmental throughput to pathway outcomes and avoidable utilization, at a time when administrative costs are estimated to account for roughly 15 to 25 percent of US national health expenditures and overall waste has been estimated at about 25 percent of spending [44]. Socially, it can be perceived as surveillance or role displacement in a workforce already under strain, with national data showing about 45 percent of physicians reporting at least one symptom of burnout in 2023 [45]. Ethically, it requires systems to prove benefit and fairness across subgroups rather than improving averages, which is challenging given evidence that widely used risk algorithms can embed substantial racial bias and meaningfully change who receives additional care when corrected. Legally, it raises questions about privacy, consent, documentation integrity, and liability, especially where algorithms influence utilization management and coverage decisions, an area already subject to explicit federal scrutiny. Operationally, it forces interoperability and cross-setting workflow redesign to reduce known failure modes such as duplicate testing associated with incomplete record transfer, which has been observed at high rates in transfer contexts. AI-WHOLE framework and operational model address these frictions by improving the data and accountability that institutions currently lack. It ties AI investments to measurable pathway outcomes and lower waste through transparent reporting, sets minimum whole-person data and interoperability requirements through procurement, requires audited workflows with named accountable owners, and mandates real-world monitoring for drift and equity so performance does not quietly degrade over time. If health systems adopt AI primarily as isolated productivity tools, the likely outcome is faster, more automated fragmentation, with inequities and downstream costs becoming harder to unwind. The near-term diffusion of AI therefore represents a narrow window to embed AI-WHOLE style governance so that these technologies function as pathway infrastructure that reduces avoidable utilization and improves function and equity at scale.

A feasible adoption strategy is pathway-centered and proceeds in three steps. First, establish an AI-WHOLE governance function with explicit accountable clinical and operational owners and apply it to 2–3 high-cost, high-variation pathways (e.g., cardiometabolic disease, heart failure, or asthma) in which improvements can be measured as reductions in preventable emergency department visits, avoidable admissions, and improved patient-reported function. Second, set a minimum whole-person data standard for these pathways and require interoperable, structured exchange through procurement, including clinical biology and omics, key behaviors, and contextual or environmental factors, so the whole person is represented explicitly rather than inferred after the fact. Interoperability policy can enable this exchange, but procurement terms and local implementation governance usually determine whether it happens in practice. Third, implement real-world evaluation and lifecycle monitoring from the beginning, including drift surveillance and stratified performance reporting, and link continuation or scale-up to prespecified thresholds for safety, effectiveness, and equity.

**Figure 1:**
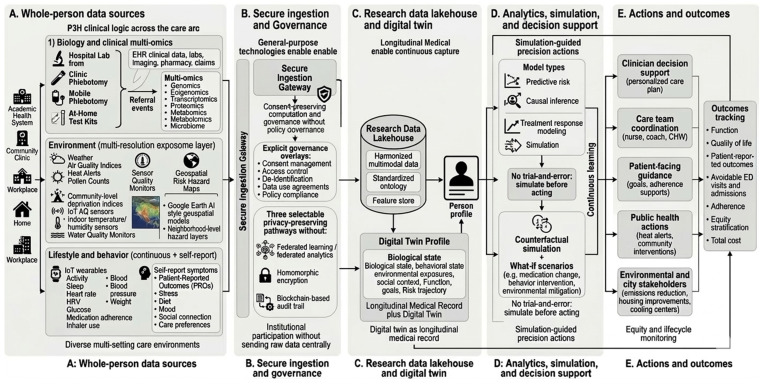
The AI-WHOLE Operating Model. An end-to-end conceptual systems diagram for precision and personalized population health. (**A**) Multi-modal data acquisition from clinical, environmental, and behavioral streams. (**B**) Secure ingestion via privacy-preserving computation (Federated Learning, Homomorphic Encryption) to maintain institutional data sovereignty. (**C**) Construction of a longitudinal Digital Twin Profile within a Research Data Lakehouse. (**D**) Simulation-guided precision actions using counterfactual modeling to optimize intervention selection. (**E**) Multi-stakeholder dissemination and a feedback loop for continuous learning, drift monitoring, and equity stratification. **Note.** This figure was generated with the assistance of Gemini 3 AI system with very detailed prompts from the authors.

To offer a practical model, we created the AI-WHOLE (Artificial Intelligence for Whole-person Health, Omics, Lifestyle, and Environment) Operating Model which represents a paradigm shift from fragmented, reactive care to a unified, proactive digital twin ecosystem (See Figure 1). Its implementation begins in Panel A (Whole-person Data Sources), where a federated data fabric populates an individual’s digital twin in real-time by standardizing multi-modal streams, integrating high-resolution environmental sensors, longitudinal multi-omics, and continuous lifestyle data into a common data model. This raw information passes through the Secure Ingestion Gateway (Panel B), ensuring that academic centers and community clinics can contribute to the global model without exporting raw data centrally, utilizing the Federated Learning pathway depicted. These streams are synthesized in the Research Data Lakehouse (Panel C) to create the Digital Twin Profile, which treats biological, behavioral, and environmental states as a single, time-aligned linkage rather than isolated variables. As illustrated in Panel D, this profile moves beyond traditional analytics to a Counterfactual Simulation module; this provides the mathematical basis for the no trial-and-error mandate, using causal inference to predict treatment responses before clinical implementation. The final stage, Actions and Outcomes (Panel E), ensures precision insights are disseminated across clinicians, patients, and public health stakeholders, while a continuous Feedback Loop returning to the initial data sources ensures the system remains self-correcting through drift monitoring and equity-focused performance checks across the patient’s lifecycle.

## 8. Conclusions

Health care’s persistent fragmentation, philosophical, operational, informational, financial, and policy-based, continues to obstruct coherent, effective, person-centered care, and it will not be solved by adding tools or exhorting better coordination. It requires an operating model in which clinical intent, scalable technologies, and governance are aligned so that biology, behavior, and environment are acted on together over time. This manuscript advances a triadic architecture to achieve that alignment. The Precision and Personalized Population Health (P3H) framework reasserts the whole person as the unit of care by organizing longitudinal action across discovery and risk identification through treatment, adherence, and sustained outcomes, while general-purpose technologies including artificial intelligence, biosensors, multimodal analytics, and scalable logistics provide the execution layer that makes continuous, low-marginal-cost care feasible beyond facility walls and across diverse settings. Policy is the decisive missing layer as without it, even high-performing technologies revert to point solutions and reproduce fragmentation. Accordingly, the AI-WHOLE policy framework (Alignment, Integration, Workflow, Holism, Outcomes, Learning, and Equity) functions as governance infrastructure that makes whole-person care implementable, measurable, and scalable by shifting accountability from isolated AI systems to end-to-end pathways, requiring explicit whole-person constructs (including goals, function, and contextual determinants), audited workflow integration with owned handoffs and escalation, and lifecycle assessment tied to outcomes that matter to patients and systems (function, preventable utilization, total cost, and equity). Importantly, whole-person health improvement often depends on stakeholders beyond clinicians and public health; environmental and transport interventions such as heat action plans and low-emission zones underscore that durable gains arise when cross-sector actors share data, coordinate workflows, and are held accountable to health outcomes alongside system efficiency. Taken together, P3H, general-purpose technologies, and AI-WHOLE reframe AI in health care from standalone applications into a governed system for whole-person care, offering a pragmatic foundation for making health care whole again.

## Figures and Tables

**Table 1:** The Seven Interdependent Phases of the P3H Framework and Corresponding GPT/AI Applications.

P3H Phase	Objective	GPTs/AI Applications	Expected Outcomes
Discovery	Identify underlying causes and risk factors of disease, integrating genomic, behavioral, and social determinants of health.	AI-driven multi-omic analysis, machine learning for disease prediction, and environmental sensing networks.	Earlier disease detection; identification of population risk clusters; improved research-to-clinic translation.
Awareness	Enhance personal and population-level understanding of risk and health status through data-driven insights.	AI chatbots for risk education, predictive health dashboards, and wearable data visualization platforms.	Enhanced health literacy and engagement; targeted risk reduction strategies at scale.
Avoidance	Prevent disease onset through early intervention and behavior modification.	Behavioral AI for lifestyle coaching, digital therapeutics, and predictive prevention analytics.	Reduced incidence of preventable diseases; cost savings from decreased disease burden.
Access	Ensure equitable, timely access to healthcare services, particularly for underserved populations.	Telehealth platforms, AI-based triage systems, and federated learning for resource-limited settings.	Increased healthcare accessibility; reduced disparities in service delivery; improved triage accuracy.
Assessment	Improve diagnostic accuracy and treatment personalization using advanced analytics.	Natural language processing for EHR synthesis, computer vision for imaging diagnostics, and multimodal data fusion.	Faster, more accurate diagnoses; precision treatment matching; reduced diagnostic errors.
Acceptance	Facilitate patient understanding, trust, and emotional readiness for care decisions.	AI-driven personalization of communication, affective computing for patient sentiment analysis, and predictive adherence modeling.	Improved patient satisfaction; higher treatment initiation and completion rates; stronger trust in clinicians and systems.
Adherence	Support continuous engagement, compliance, and outcome monitoring post-intervention.	Smart wearables, remote patient monitoring, reinforcement learning for adaptive treatment plans, and AI-assisted medication tracking.	Sustained treatment compliance; lower readmission rates; improved long-term health outcomes.

Note. This table outlines the seven interdependent phases of the Precision and Personalized Population Health Management (P3H) framework. Each phase represents a critical stage in achieving whole-person care through the integration of General Purpose Technologies (GPTs) and artificial intelligence (AI). The table demonstrates how these technologies translate the conceptual principles of P3H into measurable, actionable outcomes across the healthcare continuum.

**Table 2:** AI-WHOLE Policy Framework: Operational Definitions and Example Indicators for Real-World Measurement.

AI-WHOLE Domain	Policy Intent (What It Ensures)	Example Implementation Lever(s)	Brief Example Indicators/Performance Metrics (Illustrative)
Alignment	AI is deployed for a specified clinical purpose tied to whole-person outcomes, with accountable ownership and participatory design.	Contract and procurement requirements; clinical AI oversight committee; documented intended use; assigned ownership and accountability.	Percentage of deployments with purpose mapped to P3H stage(s); percentage with named accountable clinical and operational owners; percentage with documented co-design and sign-off; time from issue identification to governance decision (days).
Integration	Whole-person data (clinical, behavioral, social, environmental, goals/function) is interoperable, privacy-preserving, and usable across settings.	Interoperability standards; data-sharing agreements; API/HL7 FHIR requirements; privacy-by-design/federated learning architectures.	Percentage of patients with structured goals/function/social needs fields completed; cross-site data availability rate; duplicate test rate; median time to onboard a new data source into a pathway (weeks).
Workflow	AI is embedded into audited clinical pathways with defined handoffs, escalation, and human override.	Pathway certification; implementation playbooks; escalation matrices; training and competency requirements; audit trails.	Percentage of deployments with defined override and escalation; after-hours EHR time and documentation burden change; time-to-action for high-risk alerts; safety event rate attributable to AI-supported workflow (per 10,000 encounters).
Holism	Decision support represents the patient in totality and supports goal-concordant care.	Minimum data/feature standards; model input requirements; care-plan templates requiring goals/function; screening protocols.	Percentage of AI-supported decisions incorporating behavioral and contextual determinants (audited); goal-concordant care rate; documentation rate of adherence barriers (cost, transportation, literacy); referral completion rate for social/environmental needs.
Outcomes	AI is judged by patient and system results and tied to value-based goals.	Quality reporting and dashboards; payer contracts; shared savings or bundled payment models	Change in patient reported outcomes and experience and functional status; preventable ED visits and ambulatory care sensitive admissions; treatment start and adherence rates; risk adjusted total cost of care and return on investment.
Learning	AI performance is continuously monitored; drift is detected; updates are governed and transparent.	Post-deployment surveillance; drift thresholds; predetermined update protocols; model cards and reporting.	Drift detection frequency and time-to-mitigation; performance stability over time (calibration/discrimination); percentage with predefined retraining triggers and documented changes; incident reporting rate and closure time.
Equity	AI improves outcomes without widening disparities; performance is stratified and mitigation is required.	Mandatory stratified evaluation; bias audits; language accessibility; equity targets linked to contracting.	Performance parity gaps by race/ethnicity/language/geography/SES; disparity trends in access/quality/outcomes over time; subgroup false positive/negative differentials; differential uptake/adherence by subgroup; percentage with mitigation plans and demonstrated improvement against targets.

Note: Indicators are illustrative and should be selected based on the clinical pathway and local data capacity. For each domain, metrics can include implementation fidelity (process), patient/system impact (outcome), and stratified performance (equity).

## Data Availability

No new data were created or analyzed in this study. Data sharing is not applicable to this article.
